# A Smartphone App for the Management of Postoperative Home Recovery After Thoracic Surgery Procedures: A Pilot Study Using the Care4Today™ App

**DOI:** 10.3390/jcm13247843

**Published:** 2024-12-23

**Authors:** Pietro Bertoglio, Elena Garelli, Silvia Bonucchi, Jury Brandolini, Kenji Kawamukai, Filippo Antonacci, Sergio Nicola Forti Parri, Barbara Bonfanti, Giulia Lai, Lisa De Leonibus, Piergiorgio Solli

**Affiliations:** 1 Division of Thoracic Surgery, IRCCS Azienda Ospedaliero Universitaria di Bologna, 40138 Bologna, Italy; elena.garelli@aosp.bo.it (E.G.); silvia.bonucchi@gmail.com (S.B.); jury.brandolini@aosp.bo.it (J.B.); k.kawamukai@ausl.bologna.it (K.K.); filippo.antonacci@aosp.bo.it (F.A.); sergionicola.fortiparri@ausl.bologna.it (S.N.F.P.); barbara.bonfanti@ausl.bologna.it (B.B.); giulia.lai@aosp.bo.it (G.L.); lisa.deleonibus@aosp.bo.it (L.D.L.); 2 Division of Thoracic Surgery, Fondazione IRCCS Istituto Nazionale dei Tumori, 20133 Milan, Italy; piergiorgio.solli@gmail.com

**Keywords:** thoracic surgery, postoperative management, postoperative complications, re-hospitalization, smartphone

## Abstract

**Background/Objectives:** In recent years, the use of smartphones has significantly increased among populations of almost every age. The aim of our work is to analyze the impact of an application (app) that follows up with the progress of a patient who underwent a thoracic surgery procedure in the first 30 days after discharge. **Methods:** We prospectively analyzed all the patients included in the pilot study from March 2023 to September 2023. The Care4Today™ app was downloaded and activated by the patient preoperatively. From the day of discharge, the app sent questions related to pain perception, breathing capacity, general clinical conditions, problems with surgical wound and quality of life. In the case of negative responses, clinical staff received an email with an orange (medium problem) or red (serious problem) alert. **Results:** Among the 96 patients who were included, 82 eventually downloaded and used the app. The mean age of the patients was 60.7 years (range 19–80), and 43 (52.4%) were female. Minimally invasive techniques (VATS or RATS) were used in 76 cases (92.7%). The mean length of in-hospital stay was 5.3 days. Malignancy was the reason for surgery in 66 cases (80.5%). The answer rate was 75.8%. A total of 698 orange alerts and 52 red alerts were sent by the app. Re-hospitalization was needed in two cases (only one case related to our surgical procedure). The app was globally judged as useful in the management of convalescence (with an average rating of 7.4 out of 10). Age was not related to the completion rate of answers. **Conclusions:** The use of the app Care4Today could prevent unexpected re-hospitalization and possible complications. The patients appreciated the use of this tool, and they found it useful for safer postoperative recovery. No difference according to the patients’ age was found regarding the use of the app.

## 1. Introduction

In recent years, the use of smartphones has significantly increased among populations of almost every age. Modern mobile phones have evolved far beyond their original role as mere communication devices [1]. Today, a smartphone has many more functions, such as a digital camera, a pedometer, a fitness tracker and even a virtual assistant, among other capabilities. These devices have become integral to modern society, valued for their familiarity, unobtrusiveness and discretion. Equipped with numerous embedded sensors and characterized by their widespread adoption, smartphones have emerged as powerful tools across various fields of research [1]. As a matter of fact, applications (apps) and smartphones can be extremely helpful and efficient in facilitating bureaucratical paperwork and even in the management of health; for instance, smartphone apps have been described for several scopes in medicine, such as smoking cessation [2], the monitoring of psychiatric disorders [3] or increasing patients’ physical activity [4]. Furthermore, some studies explored the use of a smartphone app for the management of patients affected by lung cancer, mainly related to both preoperative rehabilitation [5] and, in the long term, the postoperative evaluation of health status and quality of life [6,7]. Nonetheless, a meta-analysis by Trifan and coworkers [8] explored medical publications in the scientific literature regarding the use of smartphone apps to monitor health, finding that there is still a lack of correlation between smartphone-generated outcomes and clinical knowledge. Moreover, on the other hand, the overuse of smartphones has been related to health problems such as visual impairment [9] and mental illness or stress increase [10].

Moreover, in the past decade, minimally invasive techniques (both Video-Assisted Thoracic Surgery [VATS] and Robot-Assisted Thoracic Surgery [RATS]) impressively spread in the medical field due to their lower impact on patients, lower postoperative pain and postoperative complication rates and reduced length of postoperative in-hospital stay [11,12]; concurrently, the dissemination of Enhanced Recovery After Surgery (ERAS) protocols [13,14] contributed to further reduce the postoperative course length. In this context, several authors proposed to further reduce the impact of surgery by using non-intubated surgery with spontaneous ventilation not only for minor procedures but also for complex surgeries [15]; similarly, some authors reported their experience of a very early removal of a chest drain on the same day of surgery for lung resections or the use of a tubeless approach for thymic surgery [16,17]. Nevertheless, despite the advantages of a fast-track approach and an early hospital discharge, the first weeks after surgery are the most critical both from physical and psychological points of view [18,19,20]. Consequently, there is an unmet clinical need for monitoring patients during the first weeks after home discharge, ensuring their needs are assessed and that any issues are addressed promptly and discreetly. The early recognition of potential problems could also help prevent unexpected outpatient visits and re-hospitalization. To address this need, a smartphone application (the Care4Today™ app, Johnson and Johnson medical, Irvine, CA, USA) was developed to monitor these patients during the first month after surgery, establishing a direct and real-time connection with clinicians to keep them informed about the patients’ conditions.

The aim of this prospective pilot study was to analyze the use of Care4Today in the first 30 days after discharge in a cohort of patients who underwent thoracic surgery procedures. We focused our analysis on the rate of moderate and severe symptoms declared by patients, the re-hospitalization rate and the unplanned outpatient visits.

## 2. Material and Methods

The present study was approved by our local ethical committee (protocol number 497/2024/Oss/AOUBo).

### 2.1. Care4Today App

In the period between March 2023 and September 2023, we ran a prospective pilot study based on the use of the Care4Today app. The app was free and was authorized by the hospital’s direction and by the privacy bureau of the University Hospital of Bologna, Italy.

The app was preoperatively downloaded by the patients who agreed to participate in this study. All patients were recognized by a personal anonymous code for privacy reasons. After self-assessing patients’ characteristics (age, body weight and smoking status) and preferred language (Italian or English) and setting the surgery date, the app started asking the patients the date of discharge from postoperative day 1 through pop-up notifications. When the patient confirmed their discharge, the app started running for 30 days. Specifically, the app sent questions daily for the first 15 days and every 4 days for the last 15 days; the questions regarded pain perception, breathing capacity, general clinical conditions, problems with surgical wound and quality of life. All questions were closed-ended, and the patients could choose either among five alternative answers or a value on a scale between 0 (worst) and 10 (best). In the event of negative responses that could indicate an actual or potential problem for the patient, an email was sent to the clinical staff highlighting an orange (moderate problem) or red (serious problem) alert; specifically, a moderate problem was considered when the answer was 4 or 5, while answers of 0, 1, 2 or 3 triggered a red alert.

At the end of the 30-day study period, the app posed two closed-ended questions to evaluate its usefulness and a global judgment (on a 0–10 scale).

The questionnaire was developed by Johnson and Johnson in collaboration with external key opinion leaders and was subjected to internal revisions from cross-functional partners. Per company policies, the list of questions in the app cannot be published.

### 2.2. Patients

In this study, we included all patients who underwent an elective thoracic surgery procedure (either lung resections or mediastinal resections), both open and minimally invasive. Patients had to be 18 year old or older, own a personal smartphone compatible with the Care4Today app and be autonomous in its use (e.g., daily use of message application). All patients were required to sign an informed consent form.

Conversely, emergency procedures or other surgeries, such as chest wall tumor resection, talc poudrage or other minor procedures, were not included in the study.

The type of intervention, the characteristics of the patients and the rate of use of the app were recorded.

### 2.3. Surgical Procedure and Patients’ Management

All patients underwent a surgical intervention under general anesthesia. Briefly, VATS procedures were performed using a biportal approach with a utility incision at the fourth or fifth intercostal space according to the type of procedure and a thoracoscopic access at the seventh or eighth intercostal space on the anterior axillary line. RATS lung resection procedures were performed using a four-arm approach: an anterior utility incision from 2.5 to 3 cm in size at the fifth or sixth intercostal space and three port incisions were made as reported elsewhere [21]. No CO_2_ inflation was used during the procedure. On the other hand, RATS thymectomy was performed using a totally endoscopic three-port approach [22] mainly using the left chest side, while right-side procedures were only used in selected cases.

Lastly, an open approach encompassed a lateral muscle-sparing thoracotomy at the fourth intercostal space.

In all procedures, a single 24 or 28 French chest drain was positioned and connected to a digital suction device (Medela, Baar, Switzerland). Although the suction policy was left to the choice of each surgeon, the chest drain was removed when the fluid output was lower than 300 mL/day and an air leak of less than 20 mL/min for at least 24 h was reported. In the case of prolonged air leak (more than five days), patients were discharged at home with a Heimlich valve and seen weekly in the outpatient clinic.

### 2.4. Statistical Analysis

Data were analyzed using the software SPSS version 26.0 for IOS (IBM, Armonk, NY, USA) and STATA 16 (StataCorp LLC, College Station, TX, USA). Continuous variables were expressed in terms of the mean with standard deviation (SD) or median with range, while categorical variables were expressed in terms of frequency. Two-tailed Pearson’s chi-square test was used for intergroup comparison of categorical variables, while Student’s t-test, an Analysis of Variance (ANOVA test) and the Mann–Whitney test were used for continuous variables.

Since the study was conducted over a fixed period of time, a proper a priori power analysis could not be performed. Therefore, we carried out a two-tailed post hoc power analysis using an effect size of 0.5 and an α error probability of 0.005, which yielded a power of 99.4%.

## 3. Results

### 3.1. Preoperative and Operative Characteristics

Among the 96 patients who were included in the study, 82 eventually downloaded and used the app (and replied to at least one question in the 30-day period).

As summarized in Table 1, the mean age of the patients was 60.7 years (range of 19–80 years), and 43 (52.4%) were female. Most of the patients underwent a pulmonary resection (64, 78.0%), and three (3.7%) underwent thymectomy. Minimally invasive techniques (VATS or RATS) were used in 76 cases (92.7%). The mean length of in-hospital stay was 5.3 days. Malignancy was the reason for surgery in 66 cases (80.5%).

### 3.2. App Results

During the whole study period, to the entire cohort, the app sent out a total of 7587 questions; in our cohort of patients, the answer rate was high, accounting for 75.8% (5753 answers).

In total, the patients reported a total of 698 moderate problems (orange alerts) and 52 severe problems (red alerts). Table 2 reports the details of all the red and orange alerts; the most frequent issues were related to pain control, followed by problems related to the wound. A large majority of orange and red alerts (96.9% and 92.5%, respectively) were treated conservatively with no need for hospitalization or unplanned outpatient visits. In six cases (7.3%), an unplanned visit was necessary, mainly for wound medication or to reassess antalgic therapy; in one case, a chest X-ray was requested as the patient complained of shortness of breath, but no abnormalities were detected, and the patient was eventually sent home. Conversely, re-hospitalization was needed in two cases (2.4%); in one case, a patient developed pericarditis after a thymectomy, while in the second case, the patient was re-hospitalized for renal colic due to the presence of a kidney stone, which required a urological procedure.

**Figure 1:**
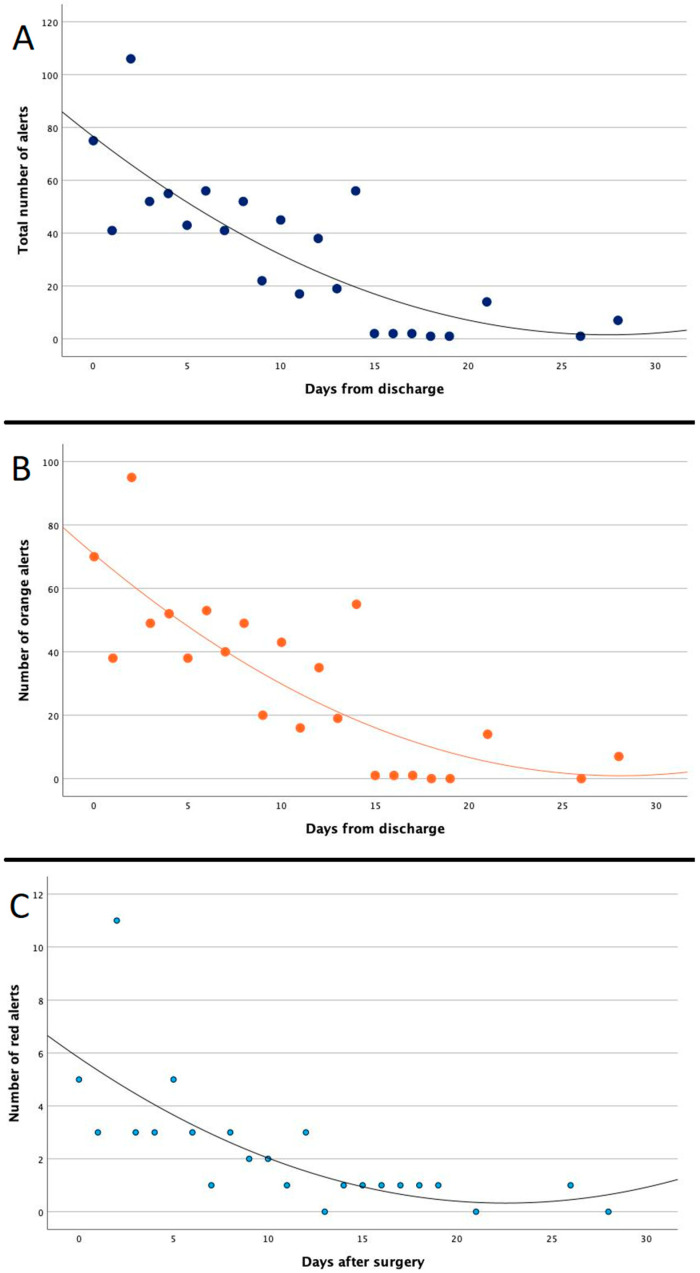
(**A**) Total number of alerts; (**B**) number of moderate (orange) alerts; (**C**) number of serious (red) alerts.

As shown in Figure 1, the numbers of orange (B) and red (C) alerts and the total number (A) of alerts were significantly higher in the first week after discharge than in the other weeks (*p* < 0.001, *p* = 0.001 and *p* < 0.001, respectively).

The global judgment and rate of the usefulness of the app in the management of the postoperative period at home were good (with an average rating of 7.8 (±2.6) out of 10 and 7.4 (±2.8) out of 10, respectively).

### 3.3. Patients Group

**Figure 2:**
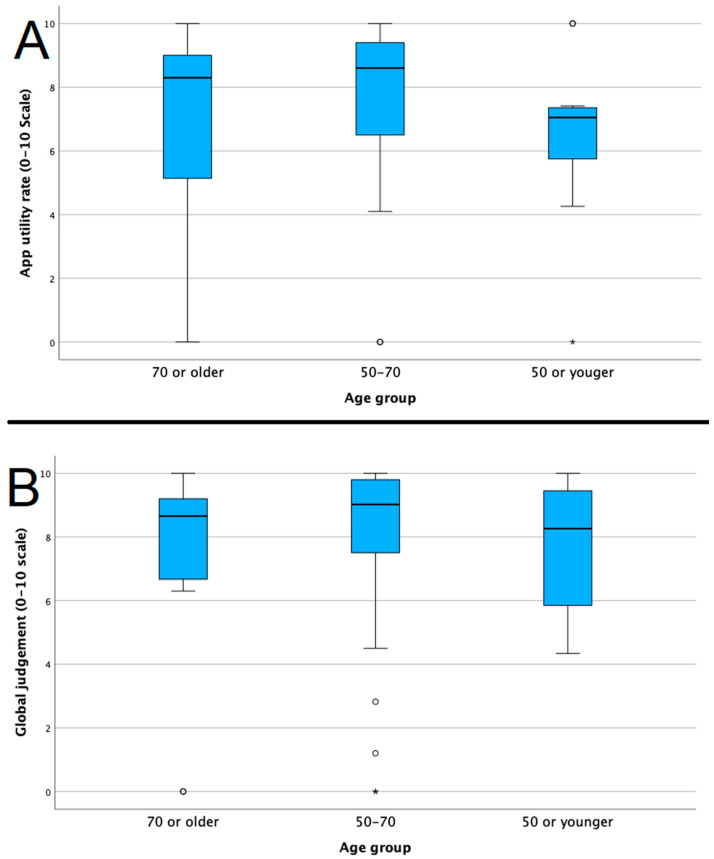
(**A**) Judgments regarding the app’s utility according to the patients’ ages. (**B**) Global judgment of the app according to the patients’ ages. Outliers are highlighted as circles for moderate outliers (1.5–3 times the interquartile range) and as asterisks for extreme outliers (beyond 3 times the interquartile range).

We did not observe differences in the answer rate according to the ages of the patients. We divided the cohort according to ages higher or equal to 70, between 50 and 70, and younger than 50. No differences were observed among the three groups in terms of the answer rate (*p* = 0.407), number of total alerts (*p* = 0.671), numbers of red alerts (*p* = 0.862) and orange (*p* = 0.626) alerts and in the final app judgments (*p* = 0.356 and *p* = 0.805; Figure 2A,B).

On the other hand, a longer in-hospital length of stay was significantly related to higher total (*p* = 0.008), orange (*p* = 0.016) and red (*p* = 0.004) alerts.

Moreover, patients who were operated on for a benign lesion judged the app to be significantly more useful compared to those affected by a malignant neoplasm (mean rate of 9.2 ± 1.2 vs. 7.4 ± 3.0; *p* = 0.039), and they had a trend towards a lower number of orange alerts (*p* = 0.061).

## 4. Discussion

New portable technologies are acquiring increasing importance in our everyday life, and they can be used for applications ranging from payment modalities to document storage. Consistently, an increasing use of smartphones has also been seen in health management in different settings. First of all, smartphones could be efficaciously used by doctors to manage and organize patients’ files and to communicate with them [23], but they could also be used to assist in medical procedures; for instance, in ophthalmology, fundoscopy can be performed with a smartphone, and it is currently considered a safe and feasible procedure that is gaining great popularity due to its portability, low costs and facility [24,25]. On the other hand, in this setting, regulatory and privacy issues remain largely unsolved. Regarding patients’ use of health monitoring apps, they have been divided into passive and active groups. The passive use of health monitoring apps, such as a pedometer, is very popular; a metanalysis by Trifan and colleagues [8] focused on the passive use of smartphones in the generation of health- and well-being-related data, and they concluded that the outcomes generated by these apps are often not related to clinical choices or improvements in clinical knowledge. Nevertheless, many studies report an active use of apps for the monitoring of specific health targets. For instance, in a meta-analysis, Xu and colleagues [26] analyzed the use of smartphone applications for the management of patients affected by arterial hypertension; the apps could help patients in different steps of their daily lives, such as by providing reminders to take medication, aiding in tracking a biometric result, providing education and motivation, and providing individualized coaching based on measured values and nonpharmaceutical behaviors. The authors concluded that the use of a health monitoring app is significantly related to reductions in blood pressure values and higher medication adherence. The use of smartphones has also been tested in several other fields, such as pulmonology [27], mental health [28,29] and psycho-oncology [30].

Smartphone apps have also been used in the management of perioperative evaluations of patients. In a Spanish study [5], the *Fissios* app was used to teach and monitor pre- and postoperative physiotherapy after elective thoracic surgery procedures; patients who used the app had lower rates of postoperative complications and shorter lengths of stay compared to those who did not. Similarly, a large European randomized controlled trial [31] compared the outcomes of elderly patients who underwent a cardiac procedure; patients who refused in-hospital intensive rehabilitation were randomized to receive a remoted-controlled home rehabilitation, and the authors found a significant benefit for the study cohort compared to the control. Nevertheless, to the best of our knowledge, no app for home postoperative monitoring after thoracic surgery procedures has been described to date.

In our study, we reported the results of a pilot study focused on the use of an app to assist patients who underwent thoracic surgery procedures after home discharge with the aims of evaluating the complication rate and re-hospitalization rate and analyzing the use of the app; the final results highlight the patients’ large interest and appreciation in using an easy technological support that could create a direct and real-time connection with the hospital and caregivers. These results might suggest that the use of this technology could further enhance fast-track protocols, reduce patients’ in-hospital stay and guarantee a safe early discharge. As a matter of fact, Kostantinidis and coworkers [32] found a significant higher proportion of readmission in patients who experienced hospitalization for longer than 7 days; on the other hand, more intuitively, Huang [20] found that readmission after a minimally invasive lobectomy was significantly related to patients’ comorbidities, while the length of stay was not. In our study, we noticed a significant correlation between a longer in-hospital stay and a higher rate of alerts, but only two patients (2.4%) required readmission in the first 30 days. Since our study was based on a different population compared to the aforementioned studies and we did not have a control group to perform a proper comparison, our re-hospitalization rate is much lower, and we might speculate that the use of the app might have prevented some re-hospitalizations by allowing for a real-time assessment of patients’ conditions.

In the experience of Huang and colleagues [20], pneumonia was the most frequent reason for readmission in the first 30 days after surgery, accounting for 19.8%; pain and wound complications were responsible of roughly 9% of readmission, similar to the rate reported by Konstantinidis [32]. In our study, the majority of alerts were related to pain perception and control and to wound problems, which could easily be managed in the majority of cases with a phone call or with an outpatient visit with no need for re-hospitalization if handled early on. As pointed out by Lei and coworkers [33], home pain management after thoracic surgery is a very challenging issue that involves both physical and psychological aspects, and it is closely related to the outcomes of the postoperative course. Moreover, a meta-analysis [34] confirmed the importance of a careful and patient-tailored approach in the management of the home postoperative course.

Although participation in our study was voluntary, we did not find any significant difference in the use of or in the evaluation of the app according to age, meaning that older patients could benefit from the use of the app as well as younger patients. A North American cross-sectional survey [35] focused on the use of technology among adults 50 years old or older that were professional or non-professional caregivers and among care recipients; the authors found that the caregivers had a significant higher use of technology compared to the care recipients, and in both groups, age was inversely proportional to the use of technologies. Although these results show that there is still a technology gap among different generations, recent evidence proves its gradual reduction [35,36].

Recently, the use of Patient-Reported Outcome Measures (PROMs) has spread as an easy and efficient way to assess patients’ quality of life not only after surgery but after all treatments. The use of PROMs in patients affected by lung cancer has been widely reported [37], and they have been significantly correlated with long-term outcomes, such as overall survival; nevertheless, one of the most critical points is still patients’ adherence to questionnaires, especially outside of clinical studies. A scoping review by Garcia Abejas and colleagues [38] focused on the use of electronic PROMs (ePROMs) to facilitate their use; the authors reported that ePROMs might simplify patients’ access and allow for a real-time evaluation of patients’ quality of life; nevertheless, privacy and security issues, together with higher costs, might limit their use. Although the Care4Today app was not designed for a proper assessment of PROMs, we might speculate that in the future, apps might be an easy tool to conduct a real-time assessment of patients.

Our study has some limitations. First of all, the number of alerts might have been influenced by the psychological statuses of the patients and might not reflect a real surgical or clinical issue. Secondly, the Care4Today app only displayed closed-ended questions, which might have reduced the possibility of patients to further explain their symptoms; moreover, the questionnaire was not validated in large cohorts. Thirdly, as already mentioned, an a priori power analysis could not be performed, and only a post hoc analysis was conducted. Additionally, the study cohort was not compared with a control cohort, and this could limit the conclusions. Lastly, we only included patients who were able to use the app autonomously, and this might have introduced some sort of selection bias.

In conclusion, we reported the results of a pilot study for the use of a smartphone app to monitor the home postoperative course of patients undergoing thoracic surgery procedures. Although this study was not designed for a comparison, in our cohort, we observed a low rate of re-hospitalization compared to the data reported in the literature that could be related to an early management of emerging problems; moreover, the use of the app was well appreciated by all patients regardless of their age.

Although wider studies are necessary to confirm our preliminary results, the use of technology seems to be able to foster the use of ERAS protocols and the early discharge of patients.

## Figures and Tables

**Table 1:** Perioperative features of the study cohort and app details.

Variable	Number
**Gender, n (%)**	
Female	43 (52.4%)
**Age mean (±SD)**	60.7 (±13.0)
**Type of surgery, n (%)**	
Lung resection	64 (78.0%)
Thymectomy	3 (3.7%)
Other	15 (18.3%)
**Lung resection, n (%)**	
Wedge resection	14 (17.1%)
Segmentectomy	17 (20.7%)
Lobectomy	33 (40.2%)
**Surgical technique, n (%)**	
Open	6 (7.3%)
Minimally invasive	76 (92.7%)
**Diagnosis, n (%)**	
Benign	16 (19.5%)
Malignant	66 (80.5%)
**Length of stay, mean (±SD)**	5.3 (±2.9)
**Answer rate, mean (±SD)**	83.2% (±15.4)
**Alert number, mean (±SD)**	
Total	9.1 (±10.8)
Orange	8.5 (±9.6)
Red	0.6 (±2.0)
**Perceived utility of app (0–10 scale), mean rate (±SD)**	7.4 (±2.6)
**Global judgment on app (0–10 scale), mean rate (±SD)**	7.8 (±2.8)

SD: Standard Deviation.

**Table 2:** Details of orange (moderate) and red (severe) alerts reported by patients.

Alert Type	Alert Description	Number
Orange	Patient has abdominal pain or pain in the shoulder	144
Orange	Patient experiences problem with wound	125
Orange	Patient has moderate pain and is taking painkillers (VAS between 6 and 8)	123
Orange	Patient has a chest pain	88
Orange	Patient is short of breath	59
Orange	Patient’s activity level is half than is was before surgery and is in pain	38
Orange	Patient has a worsening cough	35
Orange	Patient is not feeling well	35
Orange	Patient coughing up blood	14
Orange	Air escaping from the drain	10
Orange	Patient has no bowel movement on the 3rd day after discharge	7
Orange	Patient has a swollen or painful leg	6
Orange	Patient has multiple instances of dizziness	6
Orange	Patient’s wound is not dressed and not kept clean	5
Orange	Patient feels quite bad	2
Orange	Patient doesn’t understand discharge info	1
Red	Patient feels shorter of breath than normal today	20
Red	Patient has 2 out of 3 for ‘chest pain’, ‘short of breath’ and ‘swollen/painful leg’	18
Red	Patient has a lot of pain and is taking painkillers (VAS between 8 and 9)	8
Red	Patient has wound separation or a gaping wound	3
Red	Patient didn’t drink today	2
Red	Patient has multiple episodes of vomiting	1

## Data Availability

No data are shared due to privacy regulatory reasons.
